# Supplementary figures and images for: Reestablishment of spermatogenesis after more than 20 years of cryopreservation of rat spermatogonial stem cells reveals an important impact in differentiation capacity
Source: PLoS Biol. 2022 May 10;20(5):e3001618. doi: 10.1371/journal.pbio.3001618 (PMC9089916; doi:10.1371/journal.pbio.3001618)

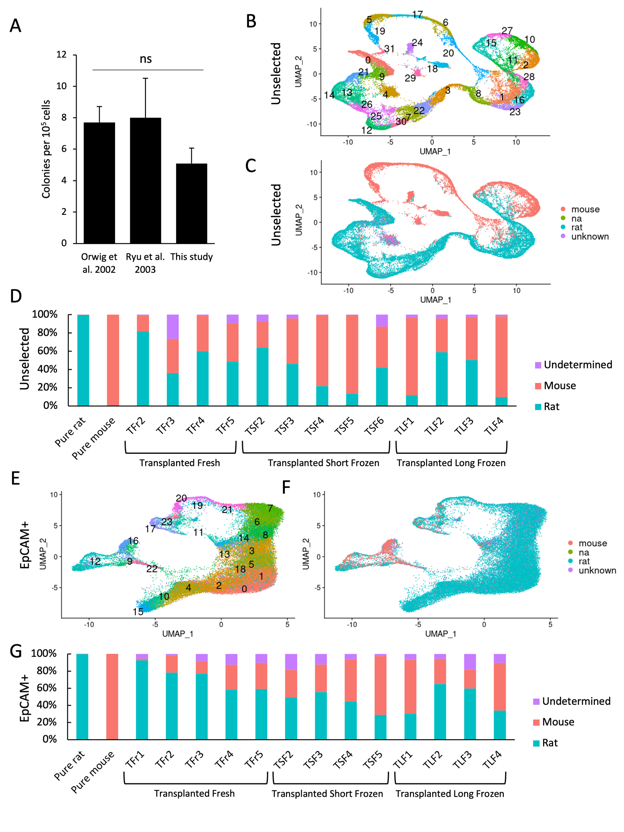

Supplement: S1 Fig — (A) Transplantation colony counts using unselected fresh rat cells are shown compared with those from studies performed approximately 20 years earlier from the same rat line and laboratory [47,48]. Novel observations shown in S4 Data. (B) UMAP projection of unbiased clustering of all unselected cells. (C) Unselected cells colored by species of origin. (D) Identity assignments of unselected rat and mouse cells in transplanted testes by alignment to both transcriptomes. Cells were assigned an identity to whichever species produced a higher number of UMI hits above a 5% threshold. Each sample represents an independent biological replicate (S4 Data). (E) UMAP projection of unbiased clustering of all EpCAM+ cells. (F) EpCAM+ cells colored by species of origin. (G) Identity assignments of EpCAM rat and mouse cells in the same manner as unselected (S4 Data). Cells with mouse or unknown identity assignments were removed from the analysis. For all UMAPs, underlying data deposited in NCBI GEO repository (GSE182438). UMAP, uniform manifold approximation and projection; UMI, unique molecular identifier. (DOCX) [file pbio.3001618.s001.docx]

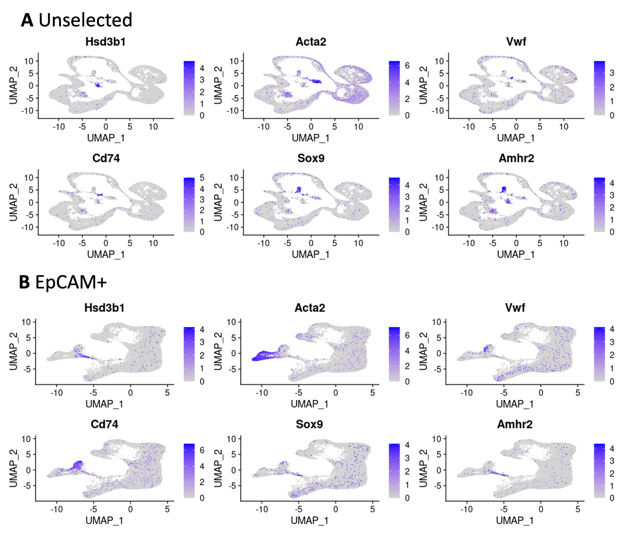

Supplement: S2 Fig — (A) Key somatic genes that identify clusters 4, 18, 24, and 29 as somatic clusters. (B) Key somatic genes that identify clusters 9, 12, 16, and 22 as somatic clusters. All somatic clusters were removed, and cells were reclustered. All underlying data deposited in NCBI GEO repository (GSE182438). UMAP, uniform manifold approximation and projection. (DOCX) [file pbio.3001618.s002.docx]

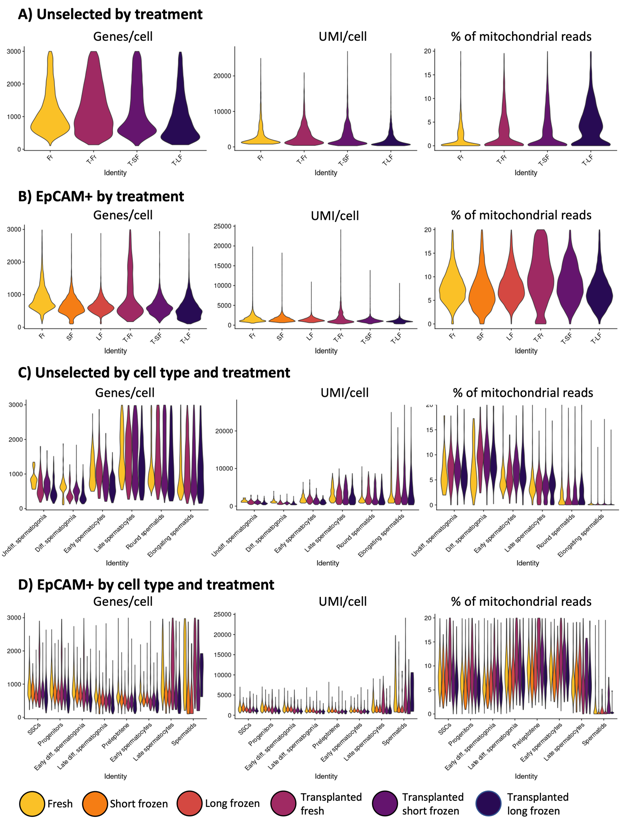

Supplement: S3 Fig — Genes/cell indicates the number of distinct genes that have one or more transcripts per cell. UMI/cell indicates the unique molecular identifier count for each cell. % mitochondrial reads shows the relative percentage of mitochondrial reads to chromosomal reads per cell. (A) All unselected cells, split by treatment. (B) All EpCAM+ cells, split by treatment. (C) Unselected cells grouped by cell type and colored by treatment. (D) EpCAM+ cells grouped by cell type and colored by treatment. All underlying data deposited in NCBI GEO repository (GSE182438). Fr, fresh; LF, long-frozen; SSC, spermatogonial stem cell; SF, short-frozen; T-Fr, transplanted fresh; T-LF, transplanted long-frozen; T-SF, transplanted short-frozen; UMAP, uniform manifold approximation and projection; UMI, unique molecular identifier. (DOCX) [file pbio.3001618.s003.docx]

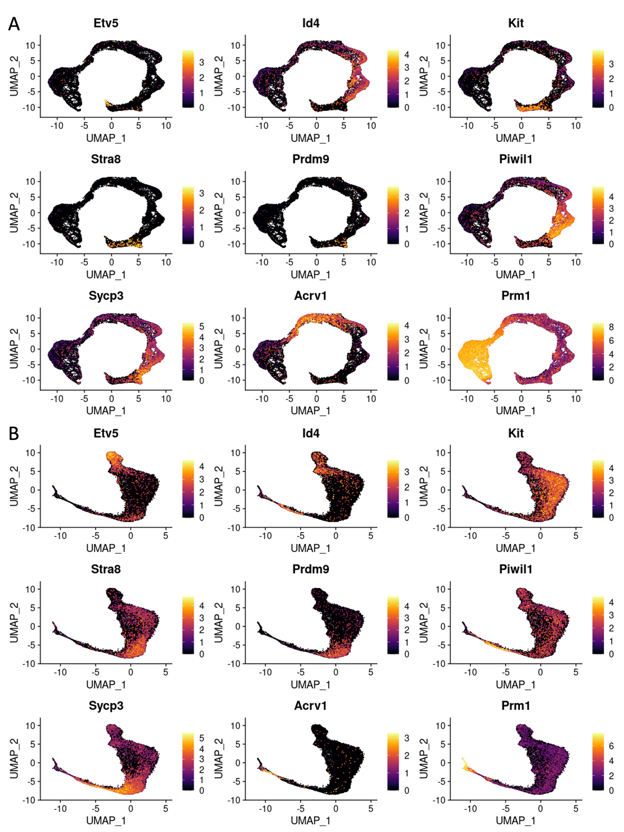

Supplement: S4 Fig — (A) UMAP projections of 9 germ cell marker genes in unselected germ cells. (B) UMAP projections of 9 germ cell marker genes in EpCAM+ germ cells. All underlying data deposited in NCBI GEO repository (GSE182438). UMAP, uniform manifold approximation and projection. (DOCX) [file pbio.3001618.s004.docx]

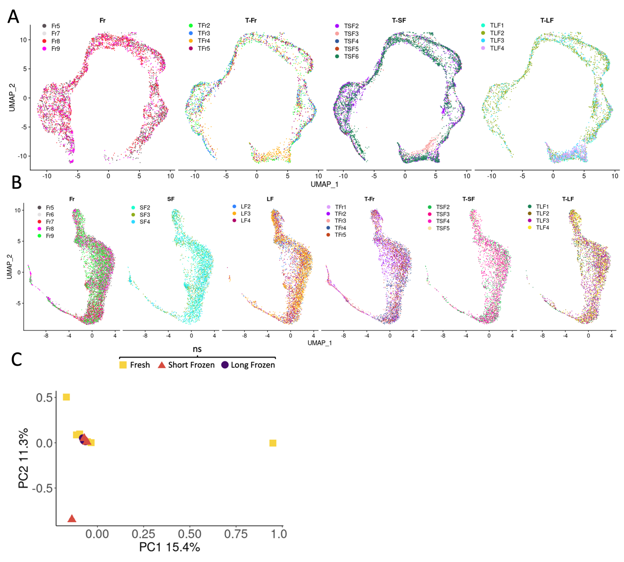

Supplement: S5 Fig — (A) All unselected cells, split by treatment and colored by replicate. (B) All EpCAM+ cells, split by treatment and colored by replicate. TLF3 and TLF4 are the same biological replicate but encapsulated on different days, otherwise each designation is a different biological replicate. (C) PCA plot derived from SNP/short indel data derived from mRNA alignments using the GATK mRNA pipeline, using the same data as Fig 4B. Percentage of variation explained by each principal component indicated on the axes. No significant difference (ns) was detected via PERMANOVA. All underlying data deposited in NCBI GEO repository (GSE182438). Fr, fresh; LF, long-frozen; PCA, principal component analysis; SF, short-frozen; SNP, single nucleotide polymorphism; T-Fr, transplanted fresh; T-LF, transplanted long-frozen; T-SF, transplanted short-frozen; UMAP, uniform manifold approximation and projection. (DOCX) [file pbio.3001618.s005.docx]

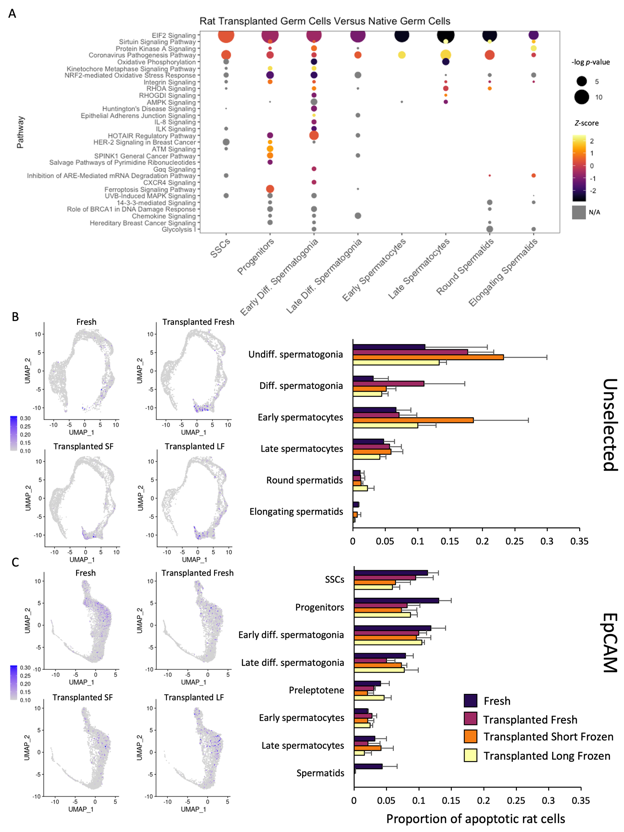

Supplement: S6 Fig — (A) Ingenuity pathway data generated from DEGs between fresh rat cells and transplanted fresh rat for each cell type. High Z-score indicates up-regulation of the pathway in fresh cells relative to transplanted. Nonsignificant Z-scores have been excluded (S4 Data). (B) An apoptosis score was generated for each cell. This was done by taking a list of proapoptotic genes (derived from Ingenuity’s database and listed below) and used Seurat’s AddModuleScore function to produce an apoptosis score for each cell. These scores are shown in the UMAP presentations (left). In addition, any cell with an apoptosis score over 0.1 (arbitrary cutoff) was designated as apoptotic, and the fraction of apoptotic cells per replicate for each cell type was calculated and the mean fraction is shown (right, S4 Data). Error bars designate SEM. (C) Above process was repeated for EpCAM+ cells. All underlying data deposited in NCBI GEO repository (GSE182438). Proapoptotic gene list: Acin1, Apaf1, Bad, Bak1, Bax, Bcl2l11, Bcl2l14, Bid, Bik, Bmf, Bnip3l, Bok, Casp2, Casp3, Casp6, Casp7, Casp8, Casp9, Casp12, Dapk1, Dapk2, Dapk3, Dedd, Dffa, Diablo, Ercc2, Ercc3, Fas, Faslg, Foxo3, Tnf, Tnfrsf10b, Tnfrsf1a, Tnfrsf1b, Tnfsf14, Tp53, Tradd, Traf3. DEG, differentially expressed gene; LF, long-frozen; SF, short-frozen; SSC, spermatogonial stem cell; UMAP, uniform manifold approximation and projection. (DOCX) [file pbio.3001618.s006.docx]

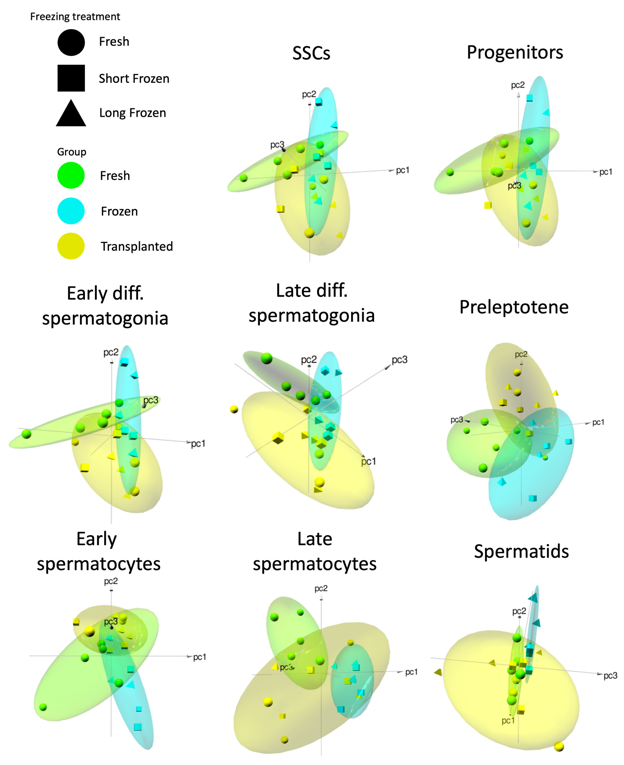

Supplement: S7 Fig — For each cell type, pseudobulked log-normalized gene expression data were used to generate principal components. 75% confidence intervals are projected for fresh samples, short-, and long-frozen grouped together and all transplanted cells as a single group. All underlying data deposited in NCBI GEO repository (GSE182438). PCA, principal component analysis; SSC, spermatogonial stem cell. (DOCX) [file pbio.3001618.s007.docx]
