# Supplementary material for: Reestablishment of spermatogenesis after more than 20 years of cryopreservation of rat spermatogonial stem cells reveals an important impact in differentiation capacity
Source: PLoS Biol. 2022 May 10;20(5):e3001618. doi: 10.1371/journal.pbio.3001618 (PMC9089916; doi:10.1371/journal.pbio.3001618)
Supplement: Attachment — Submitted filename: Whelan_response_to_reviewers.pdf [file pbio.3001618.s013.pdf]

Rev. 1:
In this paper, Brinster and colleagues transplanted long-cryopreserved rat SSCs into nude mice to find colonization of those SSCs and the production of sperm. The cryopreservation period was amazingly over 23 years. This length of time is realistic and practical when this procedure will become a clinical procedure for human patients. Thus, it is impressive and informative.

Authors, however, rather focused on a negative side of the results that such a long-preservation would affect spermatogenic competence of the SSCs, leading to defective spermatogenesis. They resorted to scRNA-seq analysis and demonstrated that long frozen sample was distinct from those of fresh and short frozen. Interestingly, the scRNA seq of transplanted samples showed that the transplanted germ cells tended to remain as SSC or spermatogonia, even when fresh sample was transplanted. This may suggest that the rat SSCs in the mouse testis had some difficulty to differentiate. It is therefore interesting to test if this would happen also when mouse SSCs were transplanted to mouse testis.

This is an exceedingly interesting question. While it is possible that rat SSCs do not differentiate with the same efficiency in the mouse environment, previous work has shown that rat spermatogenesis in the mouse appears to follow the rat differentiation program including timing being determined by the germ cells by staging (França et al 1998) and at least some tubules show quantitatively normal proportions of cells (Russell and Brinster 1996). However, the ends of tubules may be biased towards earlier stages and that might account for the difference between normal rat spermatogenesis and spermatogenesis in the transplanted animal.

Comparison with mouse to mouse transplantation would indeed be interesting, but such experiments would require many months to prepare recipients, transplant cells, then wait for spermatogenesis to regenerate.

This remains an interesting avenue to explore for our lab and we hope to address it in future work.

Franca LR, Ogawa T, Avarbock MR, Brinster RL, Russell LD. Germ cell genotype controls cell cycle during spermatogenesis in the rat. Biology of reproduction. 1998;59(6):1371-7.

Russell LD, Brinster RL. Ultrastructural observations of spermatogenesis following transplantation of rat testis cells into mouse seminiferous tubules. J Androl. 1996;17(6):615-27.

I'm not fully convinced that long-term cryopreservation, but not short-term, causes spermatogenic defects. Thus, if I'm an author of this study, I would like to change the title even though most of the text remains as it is. The title I prefer is something like these.

Revival of Spermatogenesis after over 20 years of Cryopreservation of Rat Spermatogonial Stem Cells

Resurgence of spermatogenesis after more than 20 years of cryopreservation of rat spermatogonial stem cells

We have changed the title of the manuscript to your second suggestion.

Major points:
1. It is yet difficult for me to accept the conclusion of this paper. There could be several parameters other

than long-term cryopreservation that influence the result of this study. I suspect that the quality of cryoprotectant could be critical. Quality of such reagents might have affected the result of this study.

We have made every effort to keep cryopreservation identical between the historical protocol and the current one. We used the lab notebooks for each of the cryopreserved samples and replicated the protocol, including using an ice-cream-carton-style cardboard box instead of a Mr. Frosty. Likewise, we have used the same simple cryoprotectant (i.e. 10% DMSO, 10% FBS, remainder DMEM-C). We have clarified this in the text. Lines 416-419:

> Cells were frozen in DMEM-C with final concentrations of 10% DMSO (Sigma D2650) and 10% FBS at concentrations between 1 and 4 $\times$ 10^7 cells/ml. Short frozen cells were prepared in exactly the same manner as the cryopreservation protocol used in 1996, including the freezing containers. While using the same lots of reagents was impossible, reagents were matched and of the same quality. Cell suspension was resuspended at 8 $\times$ 10^7 cells/ml in DMEM-C (DMEM with the addition of 2.2 g/L sodium bicarbonate, 100 units/ml penicillin [Life Technologies], and 100 µg/ml streptomycin [Life Technologies], 1.25 g/L sodium bicarbonate, 2 mM glutamine [Life Technologies], 0.4 mM pyruvic acid [P-5280; Sigma], 6 mM lactic acid [L-4263; Sigma], and 0.1 mM 2-mercaptoethanol [M-7522; Sigma], see reference (51)). An equal volume of 2X freezing solution made up of 20% DMSO, 20% FBS and remainder DMEM-C was prepared and added dropwise to each vial. Vials were immediately place in cardboard cartons filled with tissue paper and moved to the -80°C freezer. After 24 hours the vials were transferred to liquid nitrogen for storage. “Long-frozen” samples were stored in liquid nitrogen for 23-24 years. “Short-frozen” samples were stored for at least one month in liquid nitrogen and not more than four months.

The batch of FBS can be important for many applications and by necessity, the batches of reagents would have been different between Short and Long Frozen samples. We have added the following to the discussion to acknowledge this limitation. Lines 331-333:

> Reagents for cryopreservation media were matched to those used in the 1990s in our laboratory for the long-frozen samples, but differences in batches represents an unavoidable possible variable in an experiment spanning over 20 years.

2. It is not clear how many times the cryopreservation was performed in days of 23 years ago. In other words, how many rats were used for the cryopreservation of long frozen sample?

There were cells from 3 rats cryopreserved in 1996. Each one produced ~ 15 vials of testis cells at concentrations ranging from 1 to 4 x 10^7 cells/ml. All of the “Long Frozen” data presented in this paper is derived from these three biological replicates.

We have clarified this in the methods section. Lines 394-395:

> All long-frozen samples were derived from three original independent biological replicates.

3. In the results, it is written that stocks of Norwegian rat testis cells that were cryopreserved over 23 years ago was used in this study. On the other hand, in the materials and methods, it is written that cells from Sprague-Dawley rats were analyzed. Does this mean that different strains of rat were used in this study?

All rats used in this study were Sprague-Dawley rats from the same continuously-maintained homozygous LacZ rat line housed within our laboratory. No new rats were introduced into the colony since its inception or at any point over the study time period. We have edited the text to make this explicit.

Lines 111-114

> We designed an experiment to take advantage of stocks of Sprague-Dawley *Rattus norvegicus* testis cells that were dissociated and cryopreserved over 23 years before the start of this study (which in this paper we refer to as "long-frozen" cells).

Lines 379-382

> Cells from Sprague-Dawley rats were analyzed via scRNA-seq after the following treatments: cells freshly digested, cells frozen for a short period (<4 months) and cells frozen for a long period (>23 years). Cells for each treatment came from the same line of rats maintained continuously over the 24-year period with no addition of outside animals.

Minor points:
3. In Fig. 6B, four colors of each, light yellow, orange, dark red, and dark blue, seems to show fresh, transplanted fresh, transplanted short frozen, and transplanted long frozen, respectively. On the other hand, in 6C, the samples and the colors match in a different way. This is really a minor issue but a bit confusing.

Thank you for this observation! We have corrected Figure 6B.

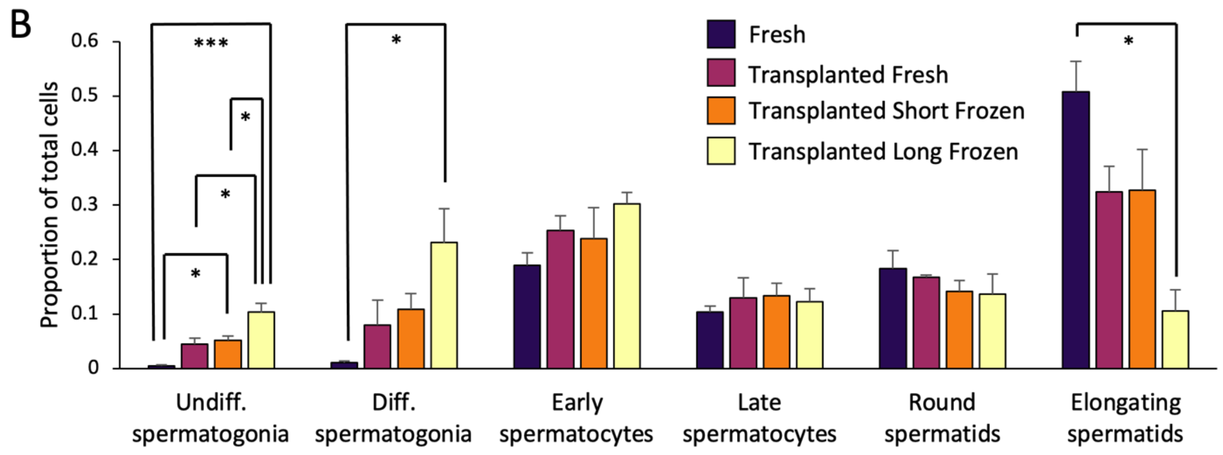

Rev. 2: Sue Hammoud – note that this reviewer has signed her review
This study by Whelan et. al. examines the molecular and functional effects of extended periods of cryopreservation on the ability of rat spermatogonial stem cells to successfully regenerate spermatogenesis. To explore this question, the authors perform a comparative scRNA-seq analysis of isolated fresh germ cell populations vs. germ cell populations isolated after transplantation. This elegant work revealed two key insights about SSC fertility preservation: First, long term crypreservation of SSCs have reduced stem cell transplantation efficiency, reduced stem cell differentiation capacity, as well as increased disorganized tubules in areas where germ cell development is restored. Second, the authors show significant molecular changes in transcriptome of fresh vs. frozen germ cells. This work is exciting and is of significant value to the reproductive biology and medicine community. Some minor concerns were noted:

1) What is the absolute frequency of tubule disorganization when comparing fresh and frozen samples? How do you distinguish between a disorganized tubule vs. sectioning vs. embedding artifact?

This is an excellent question. In order to provide a measurement of whether the cells produced colonies that completed spermatogenesis, we scored colonies (three replicates per treatment) for presence/absence of dark elongating spermatid or spermatozoan heads. This had the advantage of being an objective endpoint as they are clearly visible and also represented late differentiation stage of spermatogenesis. Therefore, we could assign a fraction of tubules completing spermatogenesis per replicate and this information is provided as a new figure, Figure 1D, and indeed Long Frozen shows a significantly smaller proportion of colonies with spermatids. See lines 132-133.

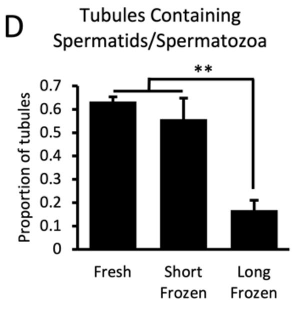

As for sectioning/embedding, we provided samples for histology in batches containing all three treatments, therefore there should be no systematic bias in processing. Figure 1E has been updated with higher quality images.

2) Can differences in stem cell activity be due to : changes in cryopreservation media composition over time. Can the differences in regenerative ability of short vs. long term frozen samples (Figure 1C) be due to certain components? Is the age of SSCs matched in fresh vs. cryopreserved samples?

We have made every effort to recapitulate the historical freezing process and used the same composition of freezing media and used the same freezing process (i.e. a cardboard box filled with tissue rather than a Mr. Frosty). We have added a limitations paragraph to the manuscript to discuss the potential for differences in the reagent lots. Lines 331-333:

Reagents for cryopreservation media were matched to those used in the 1990s in our laboratory for the long-frozen samples, but differences in batches was an unavoidable possible variable in an experiment spanning over 20 years.

The age of the rats was matched to those of the long-frozen (8-10 weeks old) for both fresh and short frozen. We have clarified this in the Methods. Lines 387-388:

Each biological replicate came from a separate animal and all rats used were age-matched to the long-frozen animals (8-10 weeks of age).

3) How does the Rat germ cell differentiation program compare to rat germ cell differentiation occurring in mouse testis? Although Rat spermatogenesis can occur in the mouse testis, no one really has explored molecular genetic differences in the differentiation program. The data is currently available in this manuscript, and only needs to be mined. Adding fresh sample data to figure 5 will be an interesting comparison?

This is an interesting comparison. The rat cells are known to establish rat characteristic spermatogenic program in the mouse, and the timing of rat spermatogenesis in the mouse is known to match rat spermatogenesis, and not mouse cycling.

Figure 5C now has Fresh samples added to the violin plots.

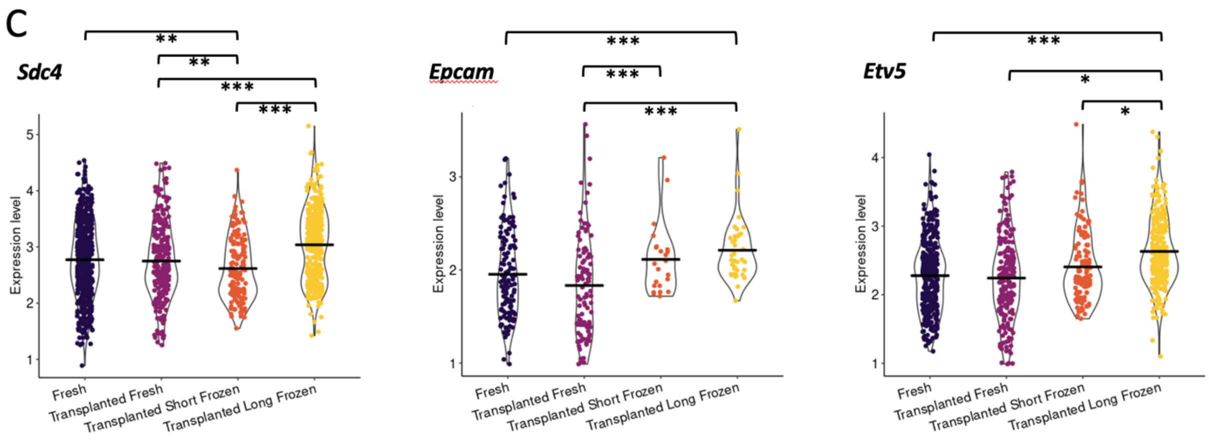

We have also replicated the Ingenuity analysis in 5D between Fresh and Fresh Transplanted treatments, which is in Supplementary Figure S6A. We have discussed this in the text, lines 228-232.

However, some differences were observed between transplanted rat cell transcriptomes as compared with cells in their native environment. Significantly perturbed pathways were concentrated largely in pre-meiotic stages, including changes in oxidative phosphorylation, oxidative stress response and DNA damage responses (Figure S6A).

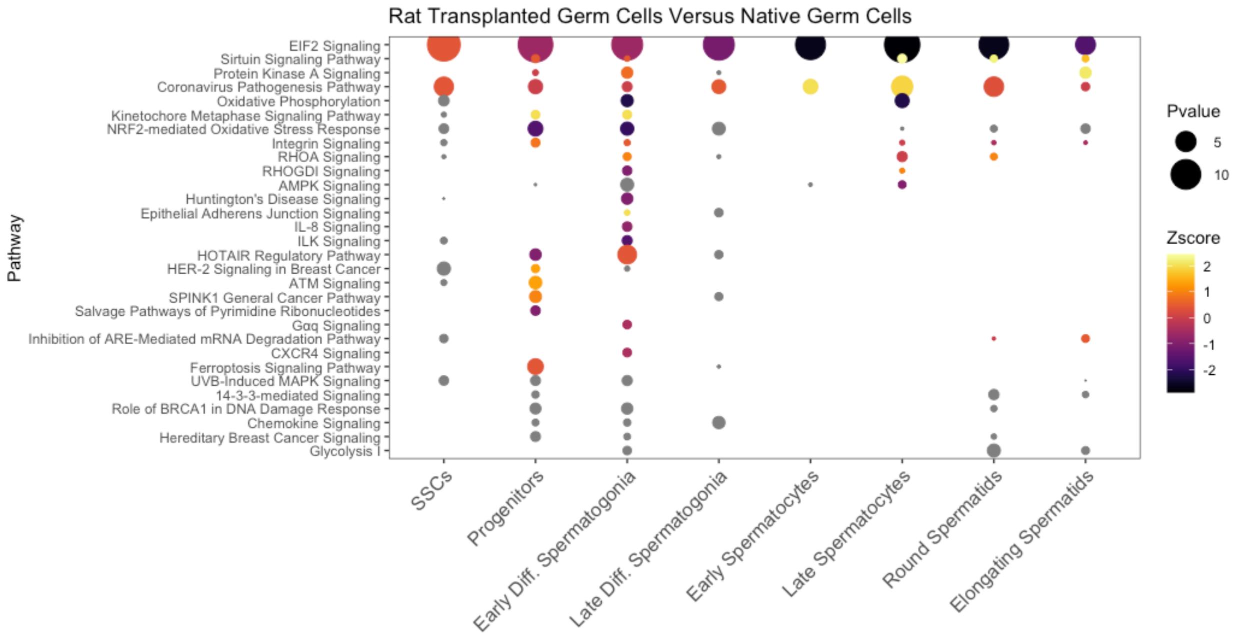

4) In Figure 6B, is it possible that the labels for the yellow and violet are swapped? The conclusion that longterm frozen cells have a higher fraction of undifferentiated cells and lower percentage of differentiated germ cells is not consistent with the data. The columns may be mislabeled.

Yes, thank you for this observation! The column colours were indeed back-to-front. We have corrected this and added a key. These colours now match between Figure 5C, 6B, 6C and the new Supplementary Figure S6.

5) Is the decrease in spermatid generation in long-term cryopreservation due to block in differentiation or increase post meiotic germ cell pruning/death?

Interesting point. There may not be a distinction – cells that fail to proceed or are in excess may undergo programmed cell death. But it is nevertheless a good question as to whether an increase in the rate of apoptosis was detectable between treatments.

We used Ingenuity's list of pro-apoptotic genes (listed in Supplementary Figure S6) and Seurat's AddModuleScore function to generate an apoptosis score for each cell. These are shown for each treatment in Supplementary Figure S6B&C along with proportions of cells displaying apoptotic markers above an arbitrary cutoff of 0.1. Clearly early cell types show higher proportions of cells displaying these markers than later stages of spermatogenesis, but no significant difference was detected between treatments.

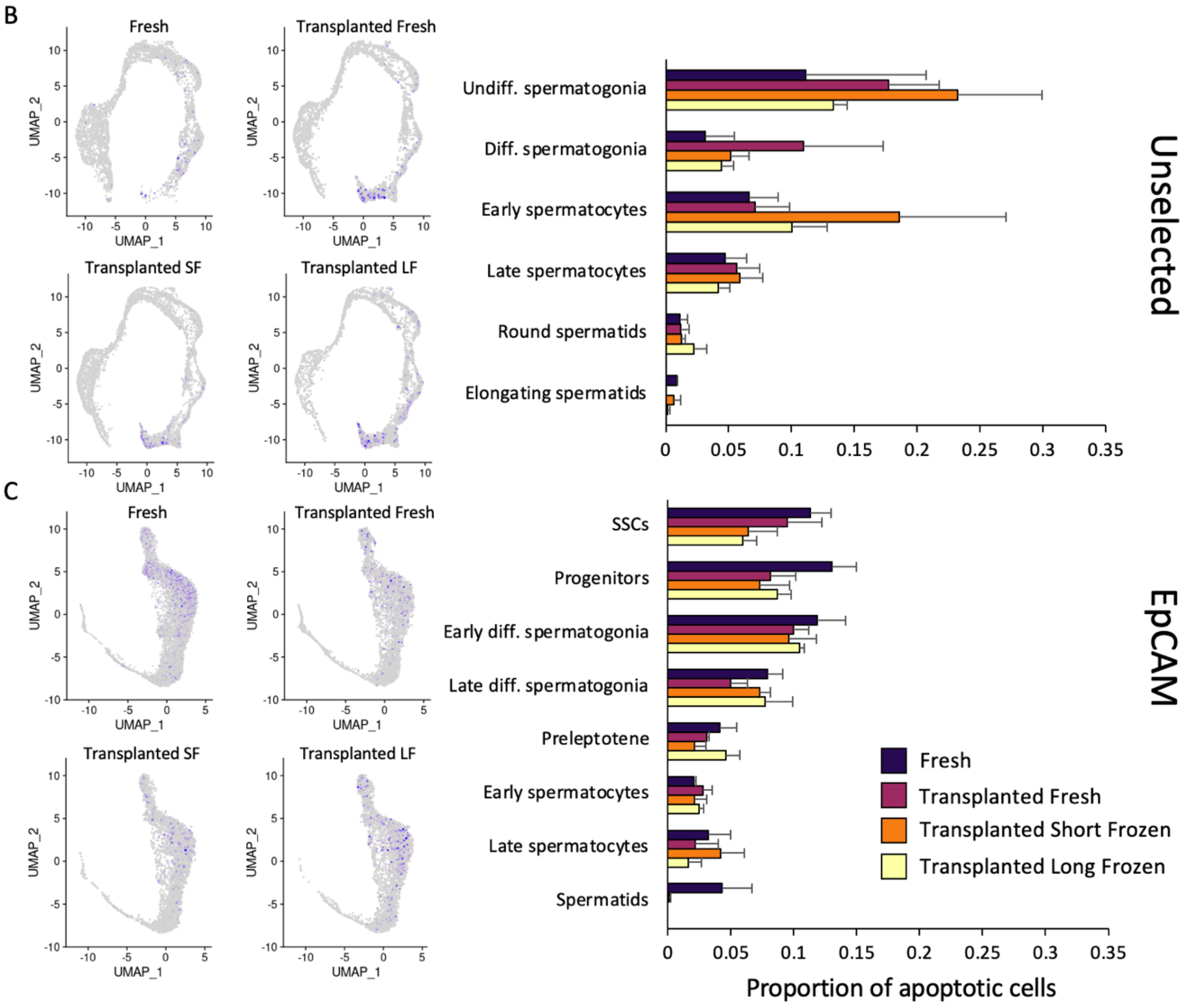
B
Fresh
Transplanted Fresh
Transplanted SF
Transplanted LF
UMAP_1
UMAP_2
Undiff. spermatogonia
Diff. spermatogonia
Early spermatocytes
Late spermatocytes
Round spermatids
Elongating spermatids
Unselected
C
SSCs
Progenitors
Early diff. spermatogonia
Late diff. spermatogonia
Preleptotene
Early spermatocytes
Late spermatocytes
Spermatids
EpCAM
Fresh
Transplanted Fresh
Transplanted Short Frozen
Transplanted Long Frozen
0 0.05 0.1 0.15 0.2 0.25 0.3 0.35
Proportion of apoptotic cells
